# Supplementary material for: A Novel Role for the Soluble Isoform of CTLA-4 in Normal, Dysplastic and Neoplastic Oral and Oropharyngeal Epithelia
Source: Cancers (Basel). 2023 Mar 10;15(6):1696. doi: 10.3390/cancers15061696 (PMC10046002; doi:10.3390/cancers15061696)
Supplement: Supplementary file 1 [file cancers-15-01696-s001.zip › cancers-2277956-supplementary.pdf]

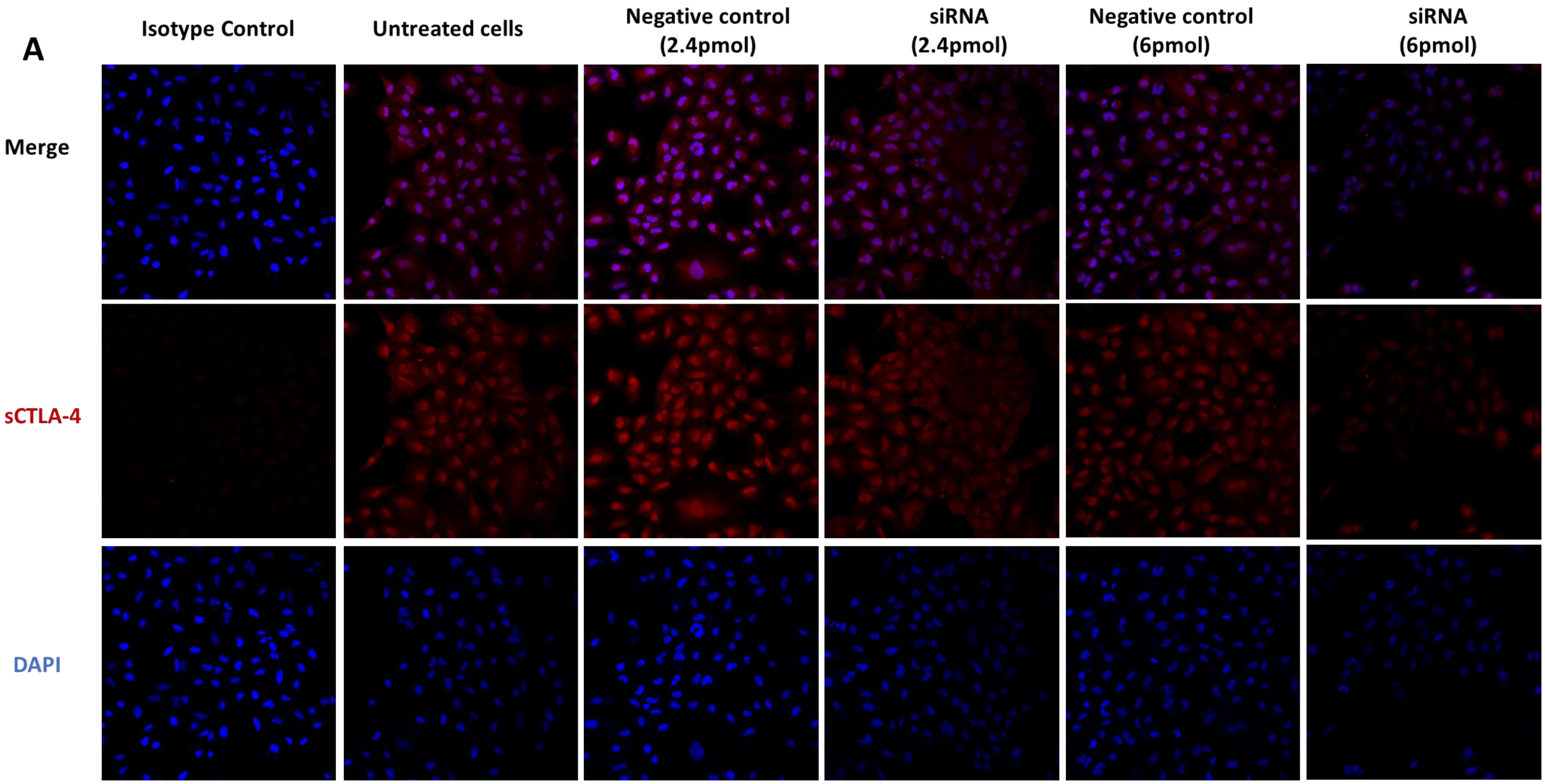

A
Isotype Control
Untreated cells
Negative control (2.4pmol)
siRNA (2.4pmol)
Negative control (6pmol)
siRNA (6pmol)
Merge
sCTLA-4
DAPI

B

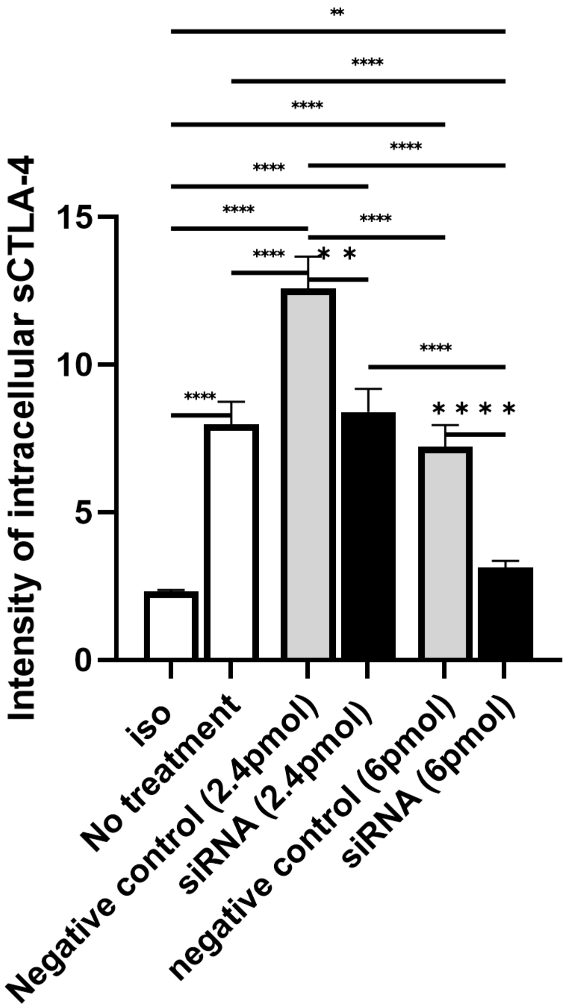

Supplementary Figure S1:siRNA was used to knockdown the CTLA-4 gene responsible for both full length and soluble isoforms in A549 lung adenocarcinoma cells to test the sensitivity of the monoclonal anti sCTLA-4 antibody 73-B1. A549 cells maintained in complete DMEM were transfected using RNAi duplex-Lipofectamine™ RNAi MAX . Two concentrations of siRNA were tested; 2.4 and 6 pmol in addition to their respective negative control. Cells were incubated for 48 hours, before staining with biotinlated 73-B1 conjugated to streptavidin-AF555 and DAPI and imaged using confocal microscopy (20x). Quantification of sCTLA-4 stanining was performed using ImageJ. A) Images show merged and separate channels of cells stained with isotype controls and with 73-B1 anti sCTLA-4 with and without different concentrations of siRNA targeting CTLA-4. B) Bar graph represents comparison of the mean intensity of sCTLA-4 in isotype control-stained cells, untreated fully-stained cells, siRNA transfected cells at two different concentrations and their respective negative controls. Error bars represent the standard error of the mean. ***P≤0.01, ****P≤0.0001,* One Way ANOVA with post-hoc Tukey test.

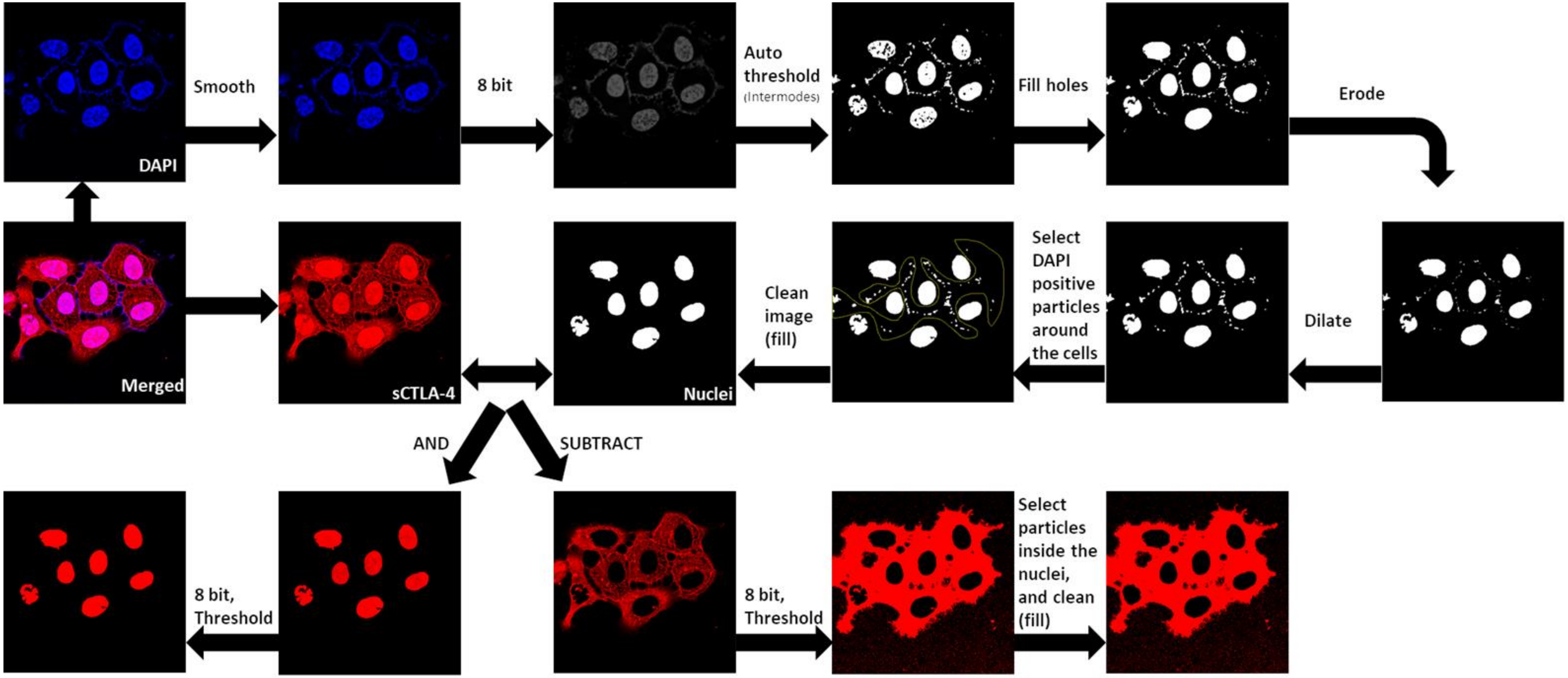

Supplementary Figure S2: A detailed schema of the image analysis process to quantify the sCTLA-4 intensity in the nucleus and cytoplasm in confocal images of three HNC cell lines. Images from merged and separate channels were obtained using Zen blue (Zeiss) and analysed using ImageJ (v 1.47v). Images of the DAPI channel (nuclei) were smoothed and converted to 8- bit grey. Auto-threshold (Intermodes) was applied resulting in a binary image in which holes were filled, followed by erosion and dilation (one iteration) and any remaining artefacts cleaned. Using the (AND) or the (SUBSTRACT) functions in the ImageJ image calculator, the sCTLA-4 channel (red) image was applied to the binary image of the nuclei to analyse the nuclei or the cytoplasm. Resulting images were converted to 8-bit grey, threshold applied (1 to 255) and particle analysis applied to analyse the mean grey value (corresponding to fluorescence intensity).

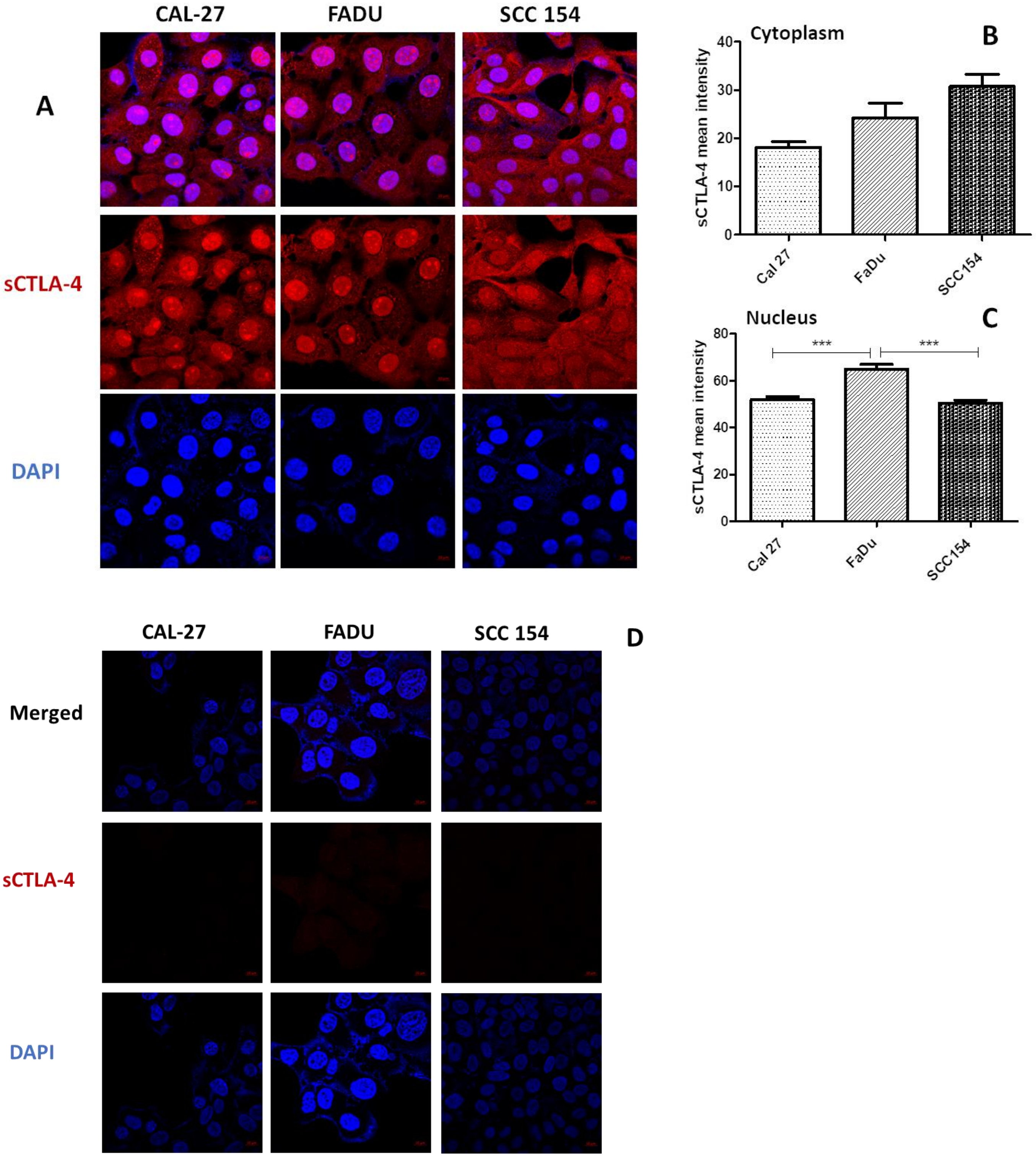

Supplementary Figure S3: Expression of sCTLA-4 in three HNC cell lines. A) merged and separate channels from CAL-27, FADU and SCC154 cell lines. B) comparison of the mean intensity of sCTLA-4 in the cytoplasm in the three cell-lines C) comparison of the mean intensity of sCTLA-4 in the nuclei of the three cell-lines. Confocal microscopy (63x), scale bars displayed at the bottom right of each image represent 10µm. ****P*≤*0.001*, Kruskal-Wallis test, Dunn's analysis. Number of images analysed CAL-27 (n=5, 63 nuclei), FADU (n=6, 57 nuclei), SCC154 (n=5, 97 nuclei). D) Isotype controls for HNC cell lines shown in figure 1. Merged and separate channels from CAL-27, FADU and SCC154 cell lines. Confocal microscopy (63x), scale bars displayed at the bottom right of each image represent 10µm.

Normal

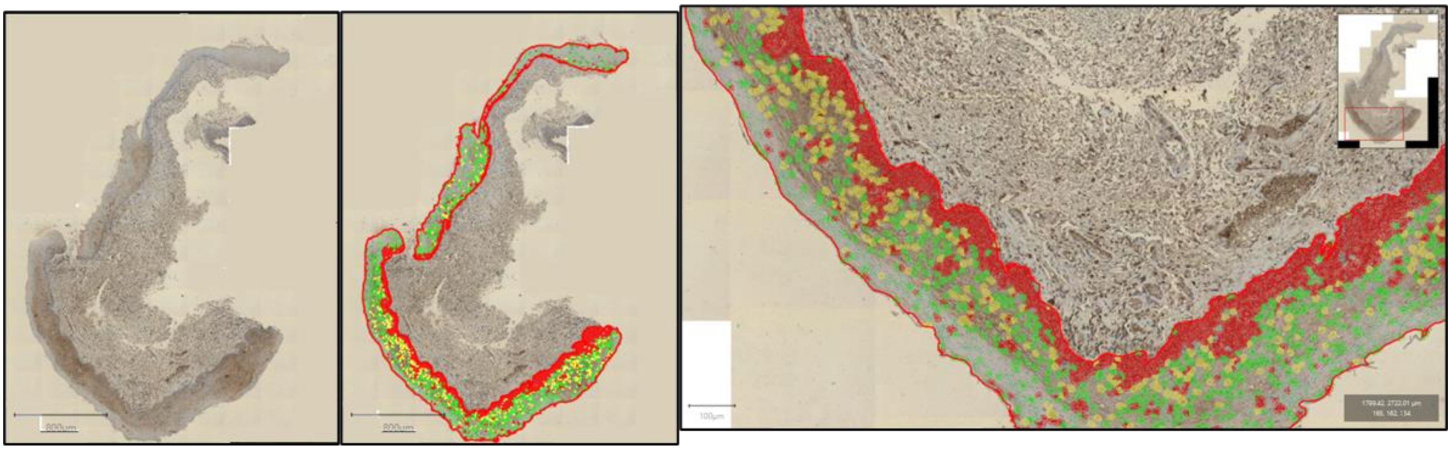

LGD

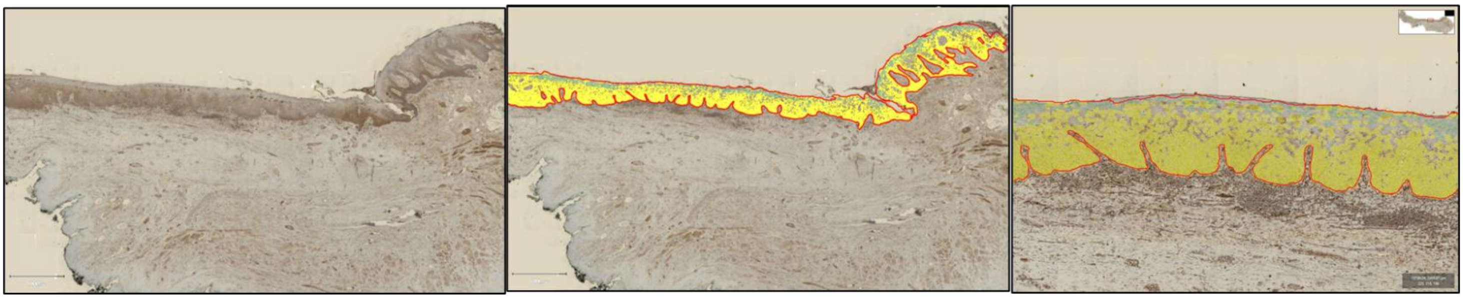

HGD

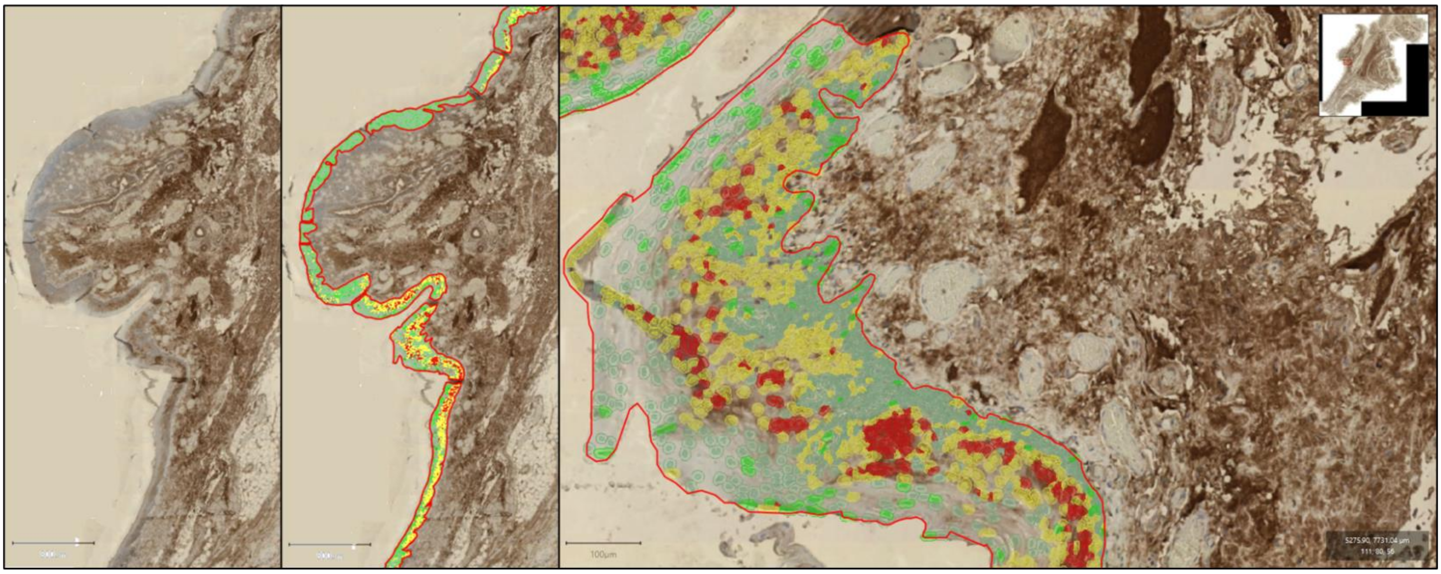

SSC

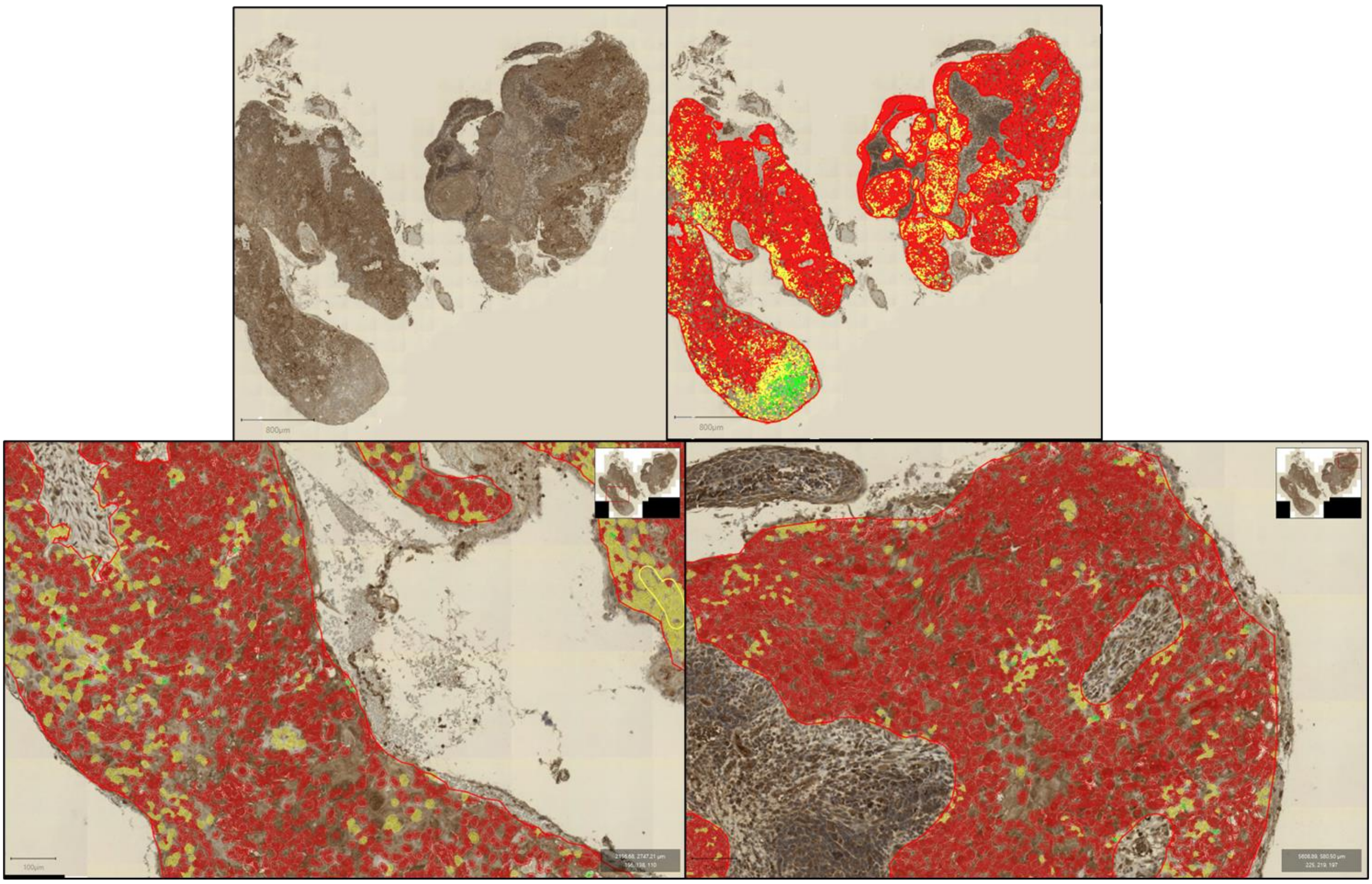

Supplementary Figure S4: More examples of representative sections from whole slide scans (x20) from normal, low-grade dysplasia (LGD), high-grade dysplasia (HGD) and squamous cell carcinoma (SCC) from the floor of the mouth.

A) Oral Cavity (floor of the mouth)

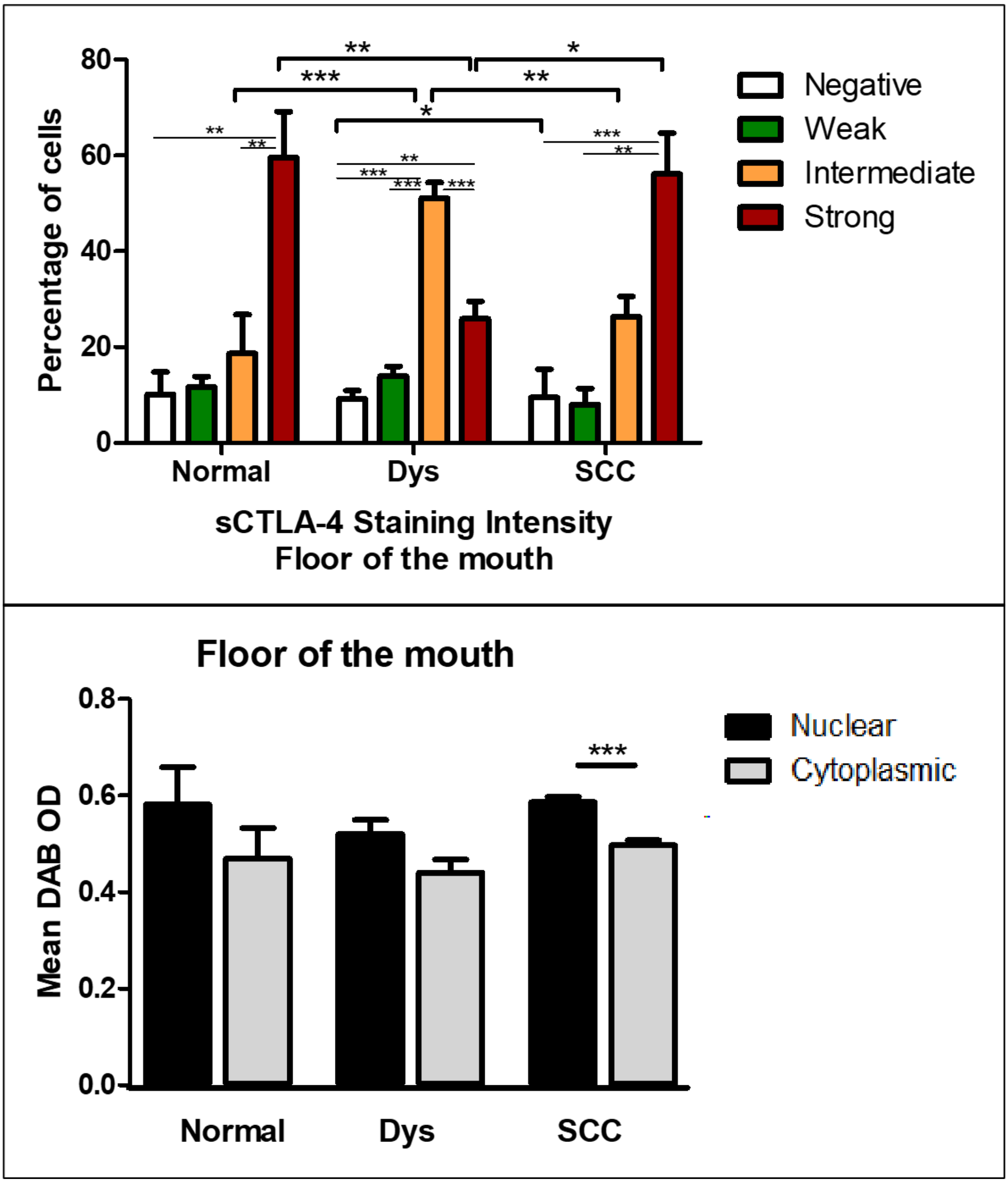

B) Oropharynx

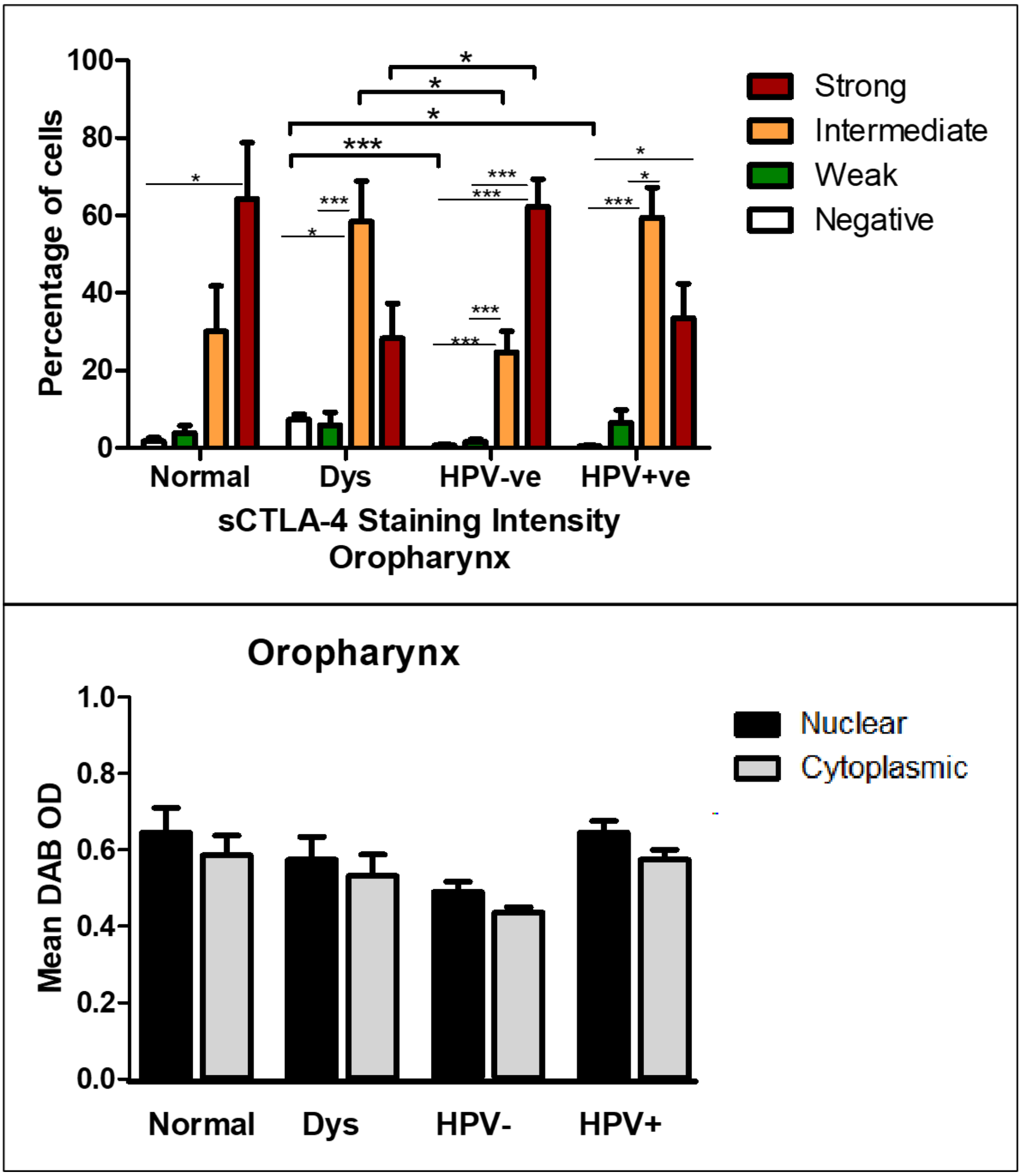

Supplementary Figure S5: A) Quantification of the staining intensity in normal, dysplasia (pooled low and high grade) and squamous cell carcinoma (SCC) from the floor of the mouth (upper panel, number of images analysed normal = 10, dysplasia= 41, and SCC= 12, Kruskal-Wallis with *post-hoc* Dunn's tests) (lower panel, number of cells analysed Normal = 32197 cells from 5 images, Dysplasia =95193 from 9 images, SCC= 186131 cells from 6 images, *t-tests*.) B) Quantification of the staining intensity in normal, dysplasia (pooled low and high grade) and HPV positive (HPV+) and HPV negative (HPV-) squamous cell carcinoma (SCC) from the oropharynx (upper panel, number of images analysed normal = 4, dysplasia= 13, HPV negative SCC= 25 and HPV positive SCC= 8, lower panel, number of cells analysed Normal = 36776 cells from 5 images, Dysplasia =72757 from 5 images, HPV-= 210150 cells from 6 images, HPV+=136423 from 8 images). Error bars represent standard error of the mean. **P< 0.05, **P< 0.01, ***P< 0.001.*

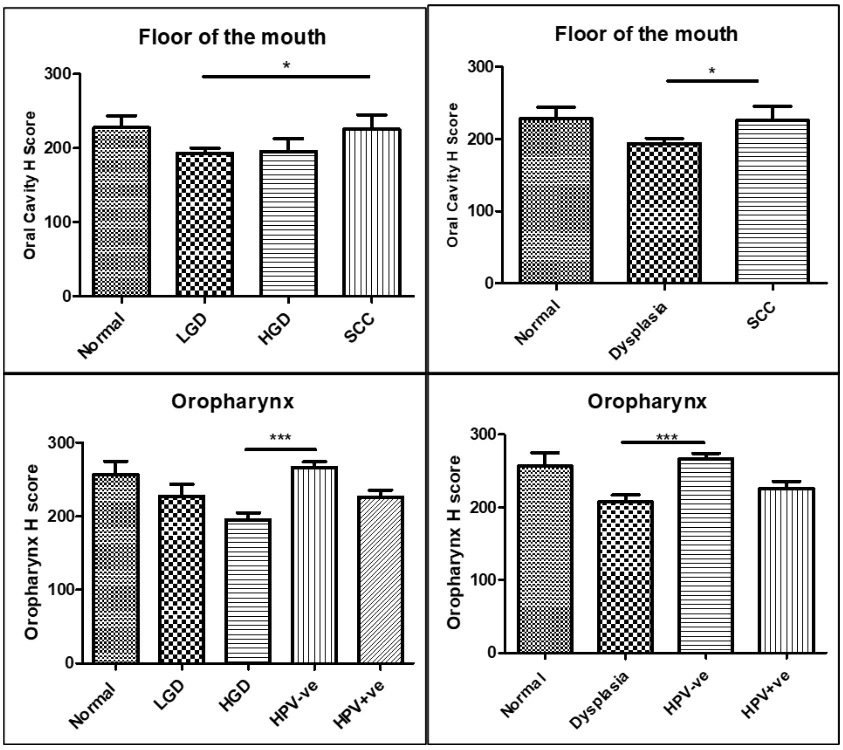

Supplementary Figure S6: H-Score for samples from the floor of the mouth (upper panel) and oropharynx (lower panel). H-Score was calculated using the formula:
H-score= 3 x percentage of strongly staining nuclei + 2 x percentage of moderately staining nuclei + percentage of weakly staining nuclei. Error bars represent standard error of the mean. **P< 0.05, ***P< 0.001,* Kruskal-Wallis with *post-hoc* Dunn's tests.

Normal

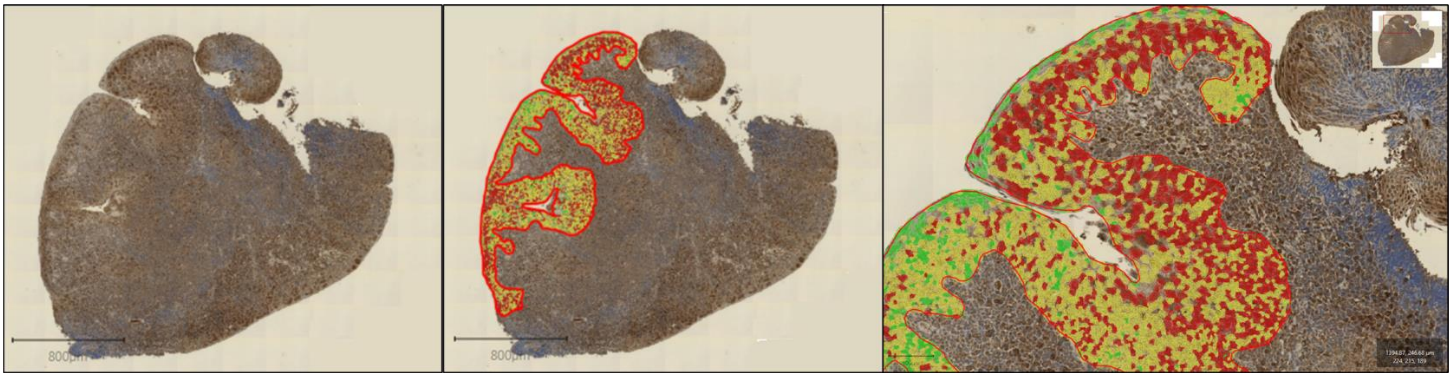

LGD

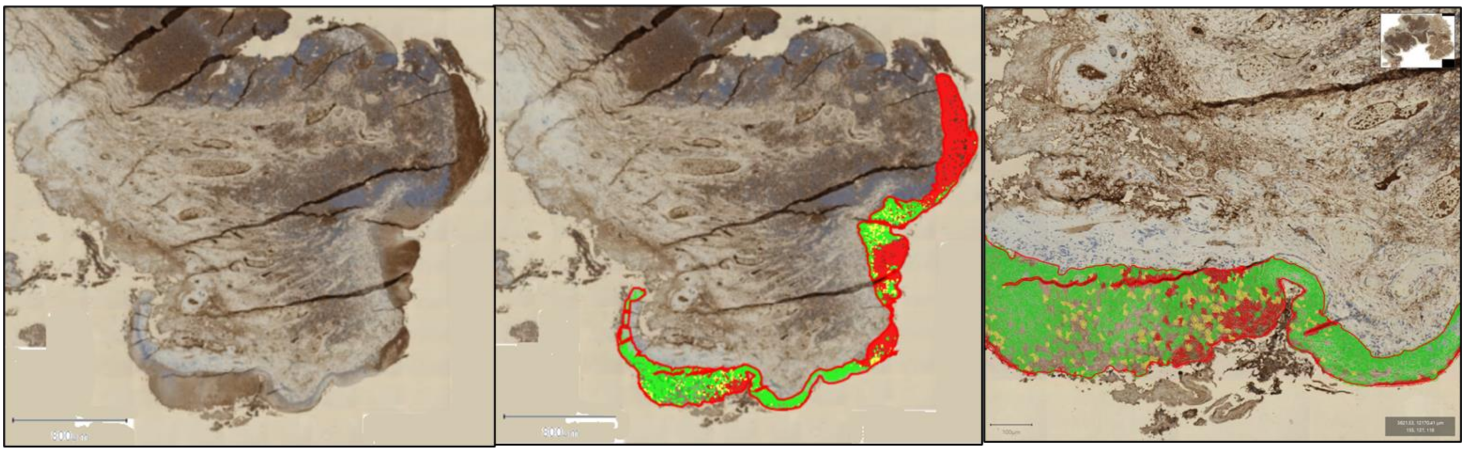

HGD

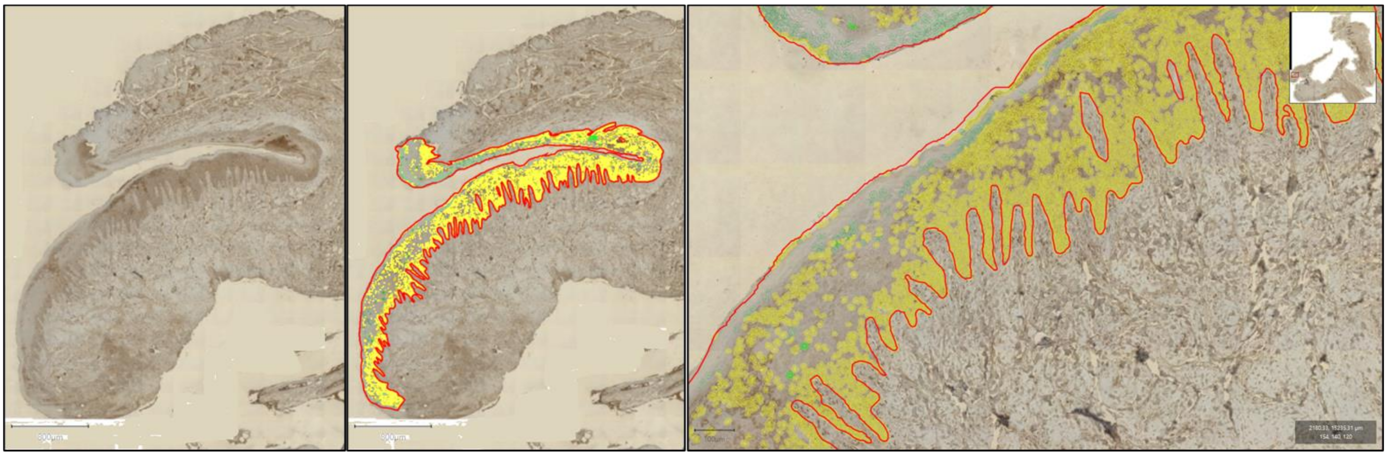

HPV-

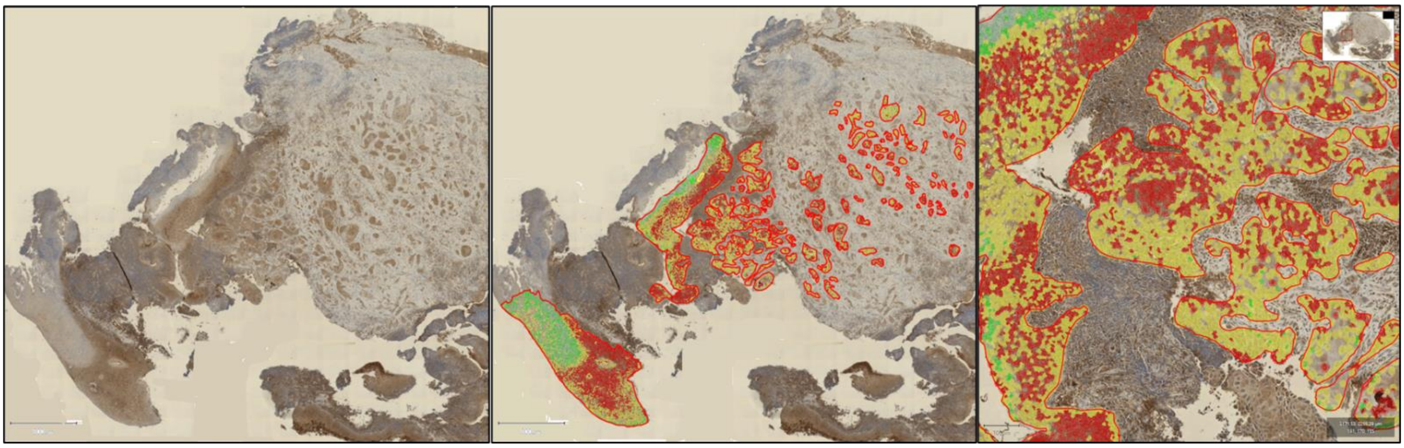

HPV+

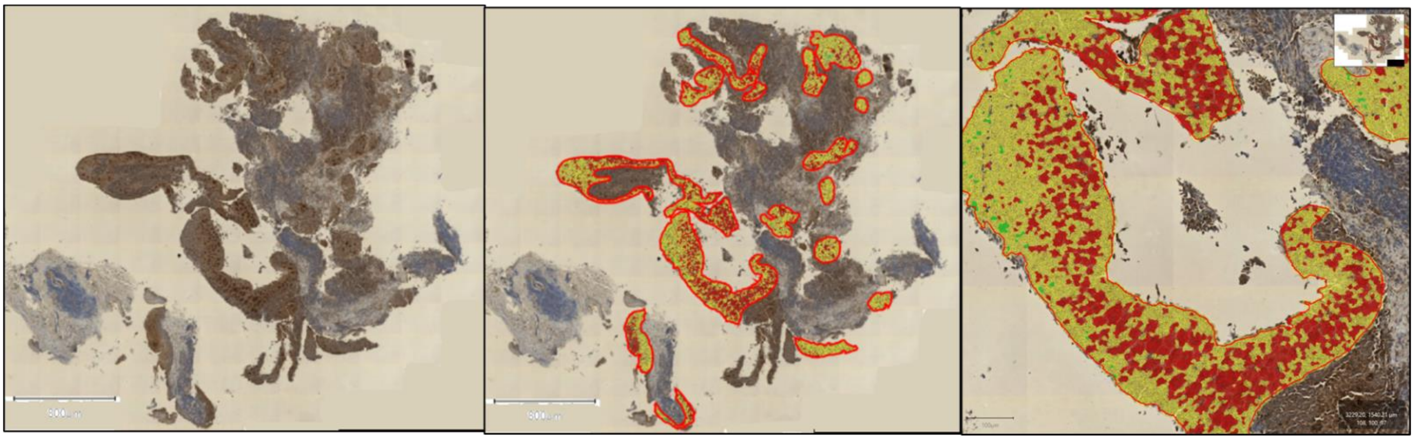

Supplementary Figure S7: More examples of representative sections from whole slide scans (x20) from normal, low-grade dysplasia (LGD), high-grade dysplasia (HGD), HPV- and HPV+ squamous cell from the oropharynx. The LGD image is a different scene from the same sample in Figure 3.
